# Functional characteristics of circulating granulocytes in severe congenital neutropenia caused by *ELANE* mutations

**DOI:** 10.1186/s12887-019-1556-x

**Published:** 2019-06-08

**Authors:** Qiao Liu, Martina Sundqvist, Wenyan Li, André Holdfeldt, Liang Zhang, Lena Björkman, Johan Bylund, Claes Dahlgren, Cai Wang, Xiaodong Zhao, Huamei Forsman

**Affiliations:** 1 Children’s hospital, Chongqing Medical University, Chongqing, China; 2 Department of Rheumatology and Inflammation Research, University of Gothenburg, Gothenburg, Sweden; 3 Rheumatology Unit, Sahlgrenska University Hospital, Gothenburg, Sweden; 4 Department of Oral Microbiology and Immunology, Institute of Odontology, Sahlgrenska Academy, University of Gothenburg, Gothenburg, Sweden

**Keywords:** SCN1, Granulocytes, Neutrophils, Eosinophils, Reactive oxygen species

## Abstract

**Background:**

Neutrophils and eosinophils are multifunctional granulocytes derived from common myelocytic-committed progenitor cells. Severe congenital neutropenia 1 (SCN1) caused by *ELANE* mutations is a rare disease characterized by very low numbers of circulating neutrophils. Little is known about the functional characteristics of the SCN1 granulocytes, except that eosinophilia has been noticed in both bone marrow and peripheral blood. In this study, we profiled the number and function of granulocytes in patients suffering from SCN1.

**Methods:**

Nine patients diagnosed with SCN1 were enrolled in this study and absolute counts of eosinophils and neutrophils from bone marrow aspirates and peripheral blood samples were analysed. In addition, Ficoll-Paque enriched granulocytes from patients and healthy controls were analysed for specific eosinophil and neutrophil markers using flow cytometry and for NADPH-oxidase activity-profile by chemiluminescence.

**Results:**

Our data demonstrate a skewed granulocyte population in SCN1 patients dominated by eosinophils in both bone marrow and peripheral blood. The latter was detected only by blood smear examination, but not by automated blood analysers. Furthermore, we show that the SCN1 eosinophils exerted normal production of reactive oxygen species generated by the NADPH-oxidase, however the response was profoundly different from that of healthy control neutrophils.

**Conclusions:**

SCN1 patients with *ELANE* mutations suffer from neutropenia yet display eosinophilia in the bone marrow and blood, as revealed by smear examination but not by automatic blood analysers. The SCN1 eosinophils are functionally normal regarding production of reactive oxygen species (ROS). However, the ROS profile produced by eosinophils differs drastically from that of neutrophils isolated from the same blood donor, implying that the eosinophilia in SCN1 cannot compensate for the loss of neutrophils regarding ROS-mediated functions.

## Background

Granulocytes (neutrophils, eosinophils and basophils) are important in the first line of host defence during microbial infections. Neutrophils comprise 55–70% of the white blood cells in circulation and execute their effects through oxygen-independent granule-stored enzymes as well as oxygen-dependent reactive oxygen species (ROS) generated by the NADPH-oxidase [1–3]. The importance of neutrophils in host defence is clearly illustrated by the fact that patients with severe congenital neutropenia (SCN) suffer from recurrent and severe infections [4–6]. SCN1 is the most common form of SCN caused by mutations in the *ELANE* gene that encodes neutrophil elastase, a serine protease synthesized at the promyelocyte stage during granulopoesis and stored in the azurophil granules [7, 8]. *ELANE* gene mutations trigger a maturation arrest of neutrophil precursors at the promyelocyte/myelocyte stage and consequently, very low absolute neutrophil counts (ANC) are found in the peripheral blood of these patients (ANC; usually < 0.5 ×  10^9^ cells/L) [9–11]. However, on many occasions, the ANC measured by automatic blood analysers is above 0.5 × 10^9^ cells/L. Absolute neutrophil counts analysed by automated blood analysers are commonly used to judge the severity of the disease and to initiate and adjust the dose for treatment with G-CSF, but whether this strategy actually is appropriate for SCN1 patients has not yet been thoroughly validated. Eosinophilia in the bone marrow and peripheral blood has been noticed in SCN [12, 13], including SCN1 patients [5, 6, 10, 14, 15]. However, very little is known about the circulating pool of eosinophils in SCN1 patients.

In this study, we characterized the granulocyte composition both in the bone marrow and in the peripheral blood through different approaches and studied the NADPH-oxidase activity of granulocytes from patients with SCN1. Our data clearly show that SCN1 patients have dramatically increased eosinophils as compared to neutrophils both in the bone marrow and in the peripheral blood. The latter was ascertained by blood smear examination, but not detected by automated blood analyser. In addition, functional analyses based on cellular NADPH-oxidase activity also confirmed that the majority of Ficoll-Paque enriched granulocytes from peripheral blood of SCN1 patients were indeed eosinophils and that these eosinophils exerted normal NADPH-activity as compared to purified eosinophils from healthy donors.

## Methods

### Participants

Peripheral blood from nine SCN1 patients, one SCN4 patient (the SCN4 patient is further described in the results section) and healthy adult controls were collected at the Children’s hospital, Chongqing Medical University, China. Informed consent was approved by the patients’ parents and controls in accordance with the Declaration of Helsinki and the study was approved by the Medical Ethics Committee of Children’s Hospital of Chongqing Medical University. Diagnosis of all patients was made by paediatricians. Two independent haematologists examined bone marrow aspirations and blood smears. Blood samples were obtained from the SCN1 patients; P01-P07 and P09 in the absence of G-CSF treatment and from P05, P06 and P08 shortly after G-CSF treatment (Filgrastim, 5–25 μg/kg/day). For each experiment, at least one healthy adult control was included and run in parallel for comparison. The experiments with purified eosinophils from healthy adult blood donors was approved by the Regional Ethical Board of Gothenburg, Sweden after informed written consent.

### Mutational analysis

Genomic DNA was extracted from peripheral blood using the QIAamp DNA Mini Kit (Qiagen, China). At least 2 μg DNA was used to construct a targeted exome library (MyGenostics, China). A final library size of 350–450 bp, including adapter sequences, was selected and 243 genes associated with primary immunodeficiency diseases and other immune-related diseases were selected by a gene capture strategy, using the GenCap custom enrichment kit (MyGenostics). All mutations identified by NextSeq 500 sequencing were confirmed by Sanger sequencing.

### Chemicals

Dextran and Ficoll-Paque were from GE-Healthcare Bio-Science (Sweden). Horseradish peroxidase (HRP) and superoxide dismutase (SOD) were from Boehringer Mannheim (Germany). Isoluminol, luminol, May-Grünwald, Giemsa, Wright’s stain and phorbol 12-myristate 13-acetate (PMA) were from Sigma (USA). The human myeloperoxidase (MPO) ELISA kit was from Immunology Consultants Laboratory Inc. (USA) and ionomycin was from Calbiochem (Germany). All flow cytometry monoclonal antibodies (mAbs) were from BioLegend (USA).

### Isolation of cells

Granulocytes and peripheral mononuclear cells (PBMCs) from peripheral blood (5 mL from patients and controls, 100 mL from healthy donors for eosinophil purification) were isolated using dextran sedimentation and Ficoll-Paque centrifugation as described [16, 17]. Purified eosinophils were obtained by subjecting the granulocyte population to negative selection using magnetic beads coated with anti-CD16 mAbs (MACS, Miltenyi Biotec Inc., USA), according to manufacturer’s instructions. After isolation, cells were kept on ice in Krebs-Ringer phosphate buffer (KRG, pH 7.3; 120 mM NaCl, 5 mM KCl, 1.7 mM KH_2_PO_4_, 8.3 mM NaH_2_PO_4_ and 10 mM glucose) supplemented with Ca^2+^ (1 mM) and Mg^2+^ (1.5 mM).

### MPO quantification, cell surface staining and NADPH-oxidase activation

Isolated cells were analysed for MPO content using a commercial ELISA kit as previously described [18]. For analysis of granulocyte composition, cells were stained with mAbs against human CD16b and CD49d, or mAbs against human CD45, CD11b, CD16, CD15, CCR3, Siglec-8, and CD14 (30 min, 4 °C) washed, and examined on a FACSCanto II (BD Biosciences). The NADPH-oxidase activity was determined using chemiluminescence (CL) [19] and measured in a six-channel Biolumat LB 9505 (Berthold Co., Germany; 1 mL system containing 1 × 10^5^ cells/mL), or a microplate reader (Synergy H1 Multi-Mode Reader, BioTek, USA; 0.2 mL system containing 3 × 10^5^ cells/mL). Intracellular CL was recorded in the presence of luminol (2 × 10^− 5^ M) and SOD (50 Units/mL), and extracellular CL was recorded in the presence of isoluminol (2 × 10^− 5^ M) and HRP (4 Units/mL) as described [19]. The CL values are presented as Mega counts per minute (Mcpm) for measurements performed in the Biolumat or relative light units (RLU) for measurements performed in the microplate reader.

### Data analysis

Data analysis was performed using GraphPad Prism 7.0a (Graphpad Software, USA), except flow cytometry data which was analysed by FlowJo 10.3 (TreeStar Inc., USA). Statistical tests used are described in the figure legends for each figure and statistical significance is indicated by *p < 0.05, **p < 0.01, ***p < 0.001, and ****p < 0.0001.

## Results

### Genetic and clinical analyses of SCN1 patients

Nine SCN1 patients (seven males and two females, age range: 1 year and 4 months – 5 years and 9 months) from eight different families (P05 and P06 are twin brothers) were enrolled in the study. A total of eight different sporadic mutations in the *ELANE* gene were identified (Table 1), with P01 and P09 harbouring novel mutations. P01 carried a deletion of CG but an insertion of A (c.669-670CG > A), resulting in a frameshift mutation (p. C223fs), and P09 harboured a G deletion (c.593del G), resulting in a premature stop codon. The ANC for all patients was equal to or below 0.5 × 10^9^/L at a minimum of at least three separate occasions during 3 months without regular cyclic fluctuations (Table 2). Immunodeficiency was apparent for all patients, as indicated by recurrent bacterial and fungal infections (Table 3). Most patients experienced ulcers free from pus at least once, further supporting the low ANC [14]. In addition, one male patient diagnosed with SCN4, age 4 years and 1 month, with compound heterozygous mutations in the *G6PC3* gene due to a stop codon in exon 2 (c.295C > T, p.Q99X) from the paternal side and a deletion in exon 6 (c.766-768del, p.256-256del) from the maternal side, was included.

**Table 1:** *ELANE* gene analyses in patients with SCN1

Patient	Sex	Age	Exon	Amino acid change^a^	Protein	Inheritance
P01	M	3y 6 m	5	c.669-670CG > A	C223fs	sporadic
P02	M	1y 5 m	4	c.377C > A	stop codon	sporadic
P03	M	1y 9 m	1	c.3G > A	p.Met1Ile	sporadic
P04	F	3y 3 m	3	c.362 T > C	p.Leu121pro	sporadic
P05	M	1y 10 m	5	c.640G > A	p.Gly214Arg	sporadic
P06	M	1y 10 m	5	c.640G > A	p.Gly214Arg	sporadic
P07	M	1y 11 m	3	c.248 T > A	p.Val83Asp	sporadic
P08	F	3y 7 m	2	c.125C > T	p.Pro42Leu	sporadic
P09	M	5y 9 m	4	c.593del G	p.C198Ffs*14	sporadic

*M* indicates Male, *F* Female, *y* Years, *m* Months

^a^All mutations were point mutations, except for P01 which was caused by two nucleotide deletions and one nucleotide insertion

**Table 2:** Laboratory parameters in blood samples from patients with SCN1

Patient	Cells, × 10^9^/L^a^					Haemoglobin^b^	CRP^c^
	WBC	Neutrophils	Eosinophils	Monocytes	Thrombocytes	(g/L)	(mg/L)
P01	6.85 (5.4–7.9)	0.78 (0.32–1.12)	0.59 (0.52–0.75)	0.77 (0.54–1.26)	710 (653–761)	96 (91–98)	19.8 (12.0–33)
P02	8.71 (5.5–11.4)	0.80 (0.50–1.14)	0.38 (0.00–0.76)	0.91 (0.21–1.82)	770 (567–1011)	109 (100–127)	30.9 (4.7–147)
P03	6.16 (3.9–8.9)	1.04 (0.00–3.90)	0.39 (0.00–0.87)	1.81 (0.03–3.87)	404 (240–702)	106 (81–123)	40.0 (0.0–161)
P04	7.79 (5.5–18.8)	0.75 (0.00–10.77)	0.32 (0.00–0.61)	0.80 (0.12–1.53)	462 (209–785)	102 (84–115)	31.4 (0.0–96)
P05	8.10 (5.9–10.7)	1.01 (0.00–2.36)	0.35 (0–00-0.79)	1.94 (0.24–3.23)	398 (243–606)	108 (90–120)	58.1 (0.5–196)
P06	7.53 (5.2–9.8)	0.91 (0.00–2.18)	0.27 (0.00–0.67)	1.35 (0.32–2.73)	415 (257–589)	104 (88–124)	59.2 (0.8–178)
P07	7.38 (4.0–11.8)	0.60 (0.20–3.10)	0.35 (0.01–0.60)	2.07 (0.71–4.13)	375 (272–737)	105 (98–111)	73.8 (13.0–157)
P08	9.60 (7.1–11.2)	0.68 (0.00–1.35)	0.74 (0.00–1.40)	1.93 (1.80–2.01)	427 (282–597)	107 (105–112)	49.6 (33.0–66)
P09	7.60 (3.2–10.2)	1.01 (0.04–3.50)	0.39 (0.03–1.13)	1.54 (0.03–4.51)	357 (237–479)	105 (94–134)	29.1 (0.8–92)

All values are the mean of > 30 measurements (range) for each patient

^a^Normal range of cell counts × 10^9^/L: White Blood cells (WBC; 4.5–15), Neutrophils (1.5–8.5), Eosinophils (0.04–0.4), Monocytes (0.1–1), and Thrombocytes (150–350) [20]

^b^Normal range of haemoglobin: 110–160 g/L

^c^Normal range of C-reactive protein (CRP): < 8 mg/L

**Table 3:** Clinical characteristics of patients with SCN1

Patient	Location of infection						Disease causing bacteria		
	Skin and soft tissue	Recurrent Pneumonia	Sepsis	Oral cavity (reccurent)	Meningitis	Recurrent ENT^a^	*Mycobacterium tuberculosis*	*Escherichia coli*	*Pseudomonas aeruginosa*
P01	Perianal abscess and Forehead cellulitis	Mild	–	+	–	+	–	+	+
P02	–	Mild	+	+	+	+	–	+	+
P03	Epicranium cellulitis and Perianal abscess^b^	Mild	–	+	+	+	–	–	+
P04	–	Severe	–	+	–	+	–	+	–
P05	Skin Cellulitis^b^	Severe	–	+	–	+	–	–	+
P06	Skin cellulitis^b^	Mild	–	+	–	+	–	–	+
P07	–	Severe	–	–	–	+	+	–	+
P08	Skin cellulitis	Severe	–	+	–	+	+	+	+
P09	Skin cellulitis	Severe	–	+	–	+	–	+	–

^a^*ENT* indicates ear, nose and throat

^b^P03, P05 and, P06 suffered from recurrent skin and soft tissue infections

### Bone marrow examinations of SCN1 patients revealed a maturation arrest of the neutrophil lineage and increased eosinophil precursors

**Fig. 1:**
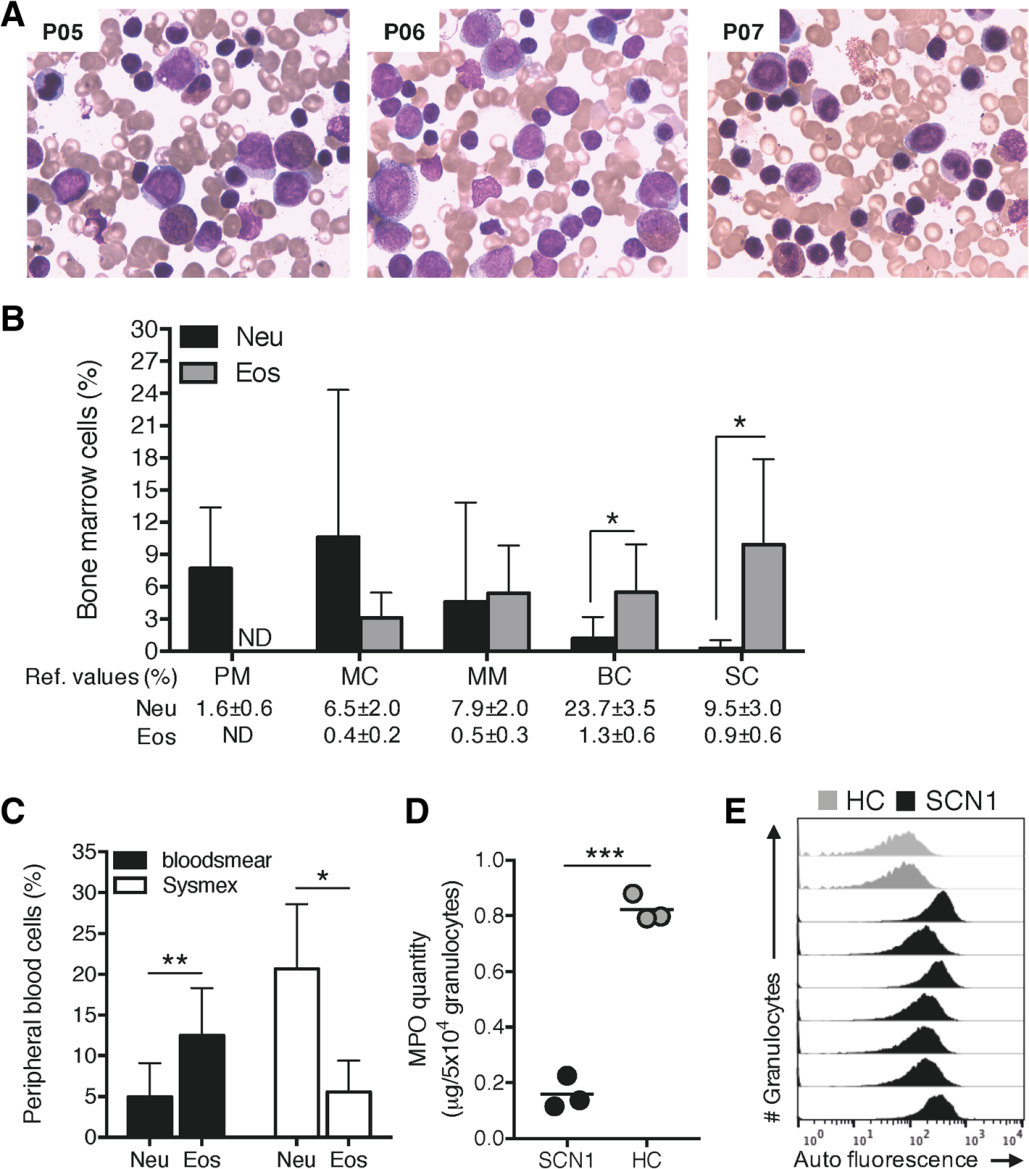
Abundance of eosinophils in bone marrow and peripheral blood from SCN1 patients. **a** Representative images of bone marrow fluid stained with Wright’s stain from three SCN1 patients (P05-P07). **b** The bar graph shows the percentage (mean + SD) of neutrophil- (Neu, black bars) and eosinophil- (Eos, grey bars) precursors during different development stages in the bone marrow; promyelocytes (PM), myelocytes (MC), metamyelocytes (MM), band cells (BC), and segmented cells (SC) from seven SCN1 patients (P01-P02 and P05-P09). Reference values are included for comparison. **c** The bar graph shows the percentage (mean + SD) of peripheral eosinophils and neutrophils generated by manual analyses of blood smears (black bars) and by automated Sysmex blood analyser (white bars) from four SCN1 patients (P01, P05-P06 and P09). **d** The concentration of MPO (μg/5 × 10^4^ granulocytes) in Ficoll-Paque enriched granulocyte lysates from three SCN1 patients (P04–06; black dots) and three healthy donors (HC; grey dots) analysed by ELISA are shown. **e** The histograms show the auto fluorescence of Ficoll-Paque enriched unstained granulocytes from two healthy controls (grey) and seven SCN1 patients (black; P02-P08) in the FITC-channel as analysed by flow cytometry. Statistical analysis in **b** and **c** was performed by paired Student’s *t*-tests comparing the percentage of neutrophils versus eosinophils (**b**) for each maturation stage separately and, (**c**) for the blood smear count and the Sysmex count separately, whilst (**d**) was analysed by an unpaired Student’s *t*-test

Bone marrow examination performed during SCN1 diagnosis revealed limited numbers of band cells (BC) and neutrophilic segmented cells (SC; Figs. 1a-b), suggesting an early-stage maturation arrest of the neutrophil lineage. Compared to reference values, the mean percentage of neutrophils precursors, promyelocytes (PM) and myelocytes (MC) were higher than the normal range (Fig. 1b). Neutrophils and eosinophils are derived from a common myelocytic-committed progenitor, the myeloblast. The presence of eosinophils in bone marrow aspirations was evident and the percentage of all eosinophil precursors were higher than the reference values (Figs. 1a-b). The number of BC and mature segmented eosinophilic SC was 5–10 times higher than the normal range, and significantly increased as compared to the BC and SC neutrophils, strongly signifying hypereosinophilia in the bone marrow of the SCN1 patients. Of note, the bone marrow examination of the SCN4 patient did not reveal any eosinophils (Figs. 5a-b).

### Automated blood analysers revealed a reduced absolute neutrophil count in the peripheral blood of the SCN1 patients

Peripheral blood counts were determined using Sysmex 800 or 2100, and data are presented as mean values for an average of > 30 blood samples per SCN1 patient collected in periods without G-CSF treatment (Table 2). The mean neutrophil numbers were reduced dramatically in all SCN1 patients as compared to the reference value (1.5–8.5 × 10^9^ /L) [20]. Yet, the mean numbers of neutrophils in patient samples, as measured with automated blood analysers, were above 0.5 × 10^9^/L, suggesting a mild disease, which contrasted the clinical manifestations (Table 3). For example, and in agreement with a previous report [6], all patient samples had increased levels of the acute phase C-reactive protein (CRP), displayed increased mean thrombocyte counts (higher than the normal range), and also high monocyte and eosinophil counts (above or at the upper level of the normal range), indicating frequent infections/inflammation. Of note, the mean eosinophil counts did not exceed the mean neutrophil counts for any patient, except for P08 (Table 2). Also, automated blood analysis of the SCN4 patient revealed reduced neutrophil counts (mean 0.78 × 10^9^/L) but normal eosinophil counts (mean 0.06 × 10^9^/L; data not shown).

### Blood smear examination demonstrated higher eosinophil counts than neutrophil counts in the circulation of SCN1 patients

The mean ANC (> 0.5 × 10^9^/L) and eosinophils from the automated blood analysers (Table 2) did not correlate with the number of mature neutrophils and eosinophils in the bone marrow (Figs. 1a-b) and was in disagreement with the disease severity (Table 3) [15]. Hence, we next performed blood smears to manually determine the granulocyte composition from four SCN1 patients and in parallel, these samples were again analysed by automated blood analysers. A large discrepancy was noticed regarding neutrophil and eosinophil counts obtained by these two methods. The blood analysers revealed significantly higher neutrophil numbers than eosinophils, whereas the blood smear examination demonstrated the opposite, i.e., significantly higher eosinophil numbers than neutrophils (Fig. 1c). The eosinophil counts obtained by manual counting of blood smears were also higher than the reference values for eosinophils (1–5% [21]), indicating eosinophilia in the blood of SCN1 patients. In summary, the data revealed by blood smear examination but not automated blood analysers clearly showed increased eosinophils in peripheral blood of the SCN1 patients, and agreed not only with the bone marrow examination but also with the clinical manifestations of the patients.

### MPO quantity and flow cytometry revealed that the granulocyte fractions of the SCN1 blood were dominated by eosinophils and not neutrophils

To further characterize the SCN1 patients’ granulocytes, we utilized the Ficoll-Paque separation method [16, 17]. Using 5 mL of blood from the SCN1 patients, Ficoll-Paque separation recovered granulocytes in numbers ranging from 0.1 × 10^6^ cells (P02) up to 3.76 × 10^6^ cells (P05 and P06 after G-CSF treatment). All experiments were performed on granulocytes from patients without G-CSF treatment if not indicated. To elucidate the presence of neutrophils and eosinophils in these granulocyte samples, we first screened for neutrophils by measuring MPO in P04, P05 and P06. MPO is a peroxidase abundantly present in neutrophil azurophil (primary) granules and monocytes, but not in eosinophils. The MPO levels in the SCN1 granulocytes were significantly decreased as compared to granulocyte samples from healthy controls (Fig. 1d), indicating that the SCN1 samples contained low amounts of neutrophils. We next tested for the presence of eosinophils in the Ficoll-Paque enriched granulocyte samples, by taking advantage of the fact that eosinophils are highly auto fluorescent compared to neutrophils when excited by the argon 488-laser and measured in the Fluorescein (FITC)-channel by flow cytometry [22, 23]. Compared with control granulocytes, SCN1 granulocytes were noticeably more auto fluorescent (Fig. 1e), which together with the reduced MPO levels strongly suggested more eosinophils than neutrophils in the SCN1 patients’ Ficoll-Paque enriched granulocytes as compared to controls. Of note, no increased auto fluorescence was seen in granulocytes isolated from the SCN4 patient (Fig. 5c).

**Fig. 2:**
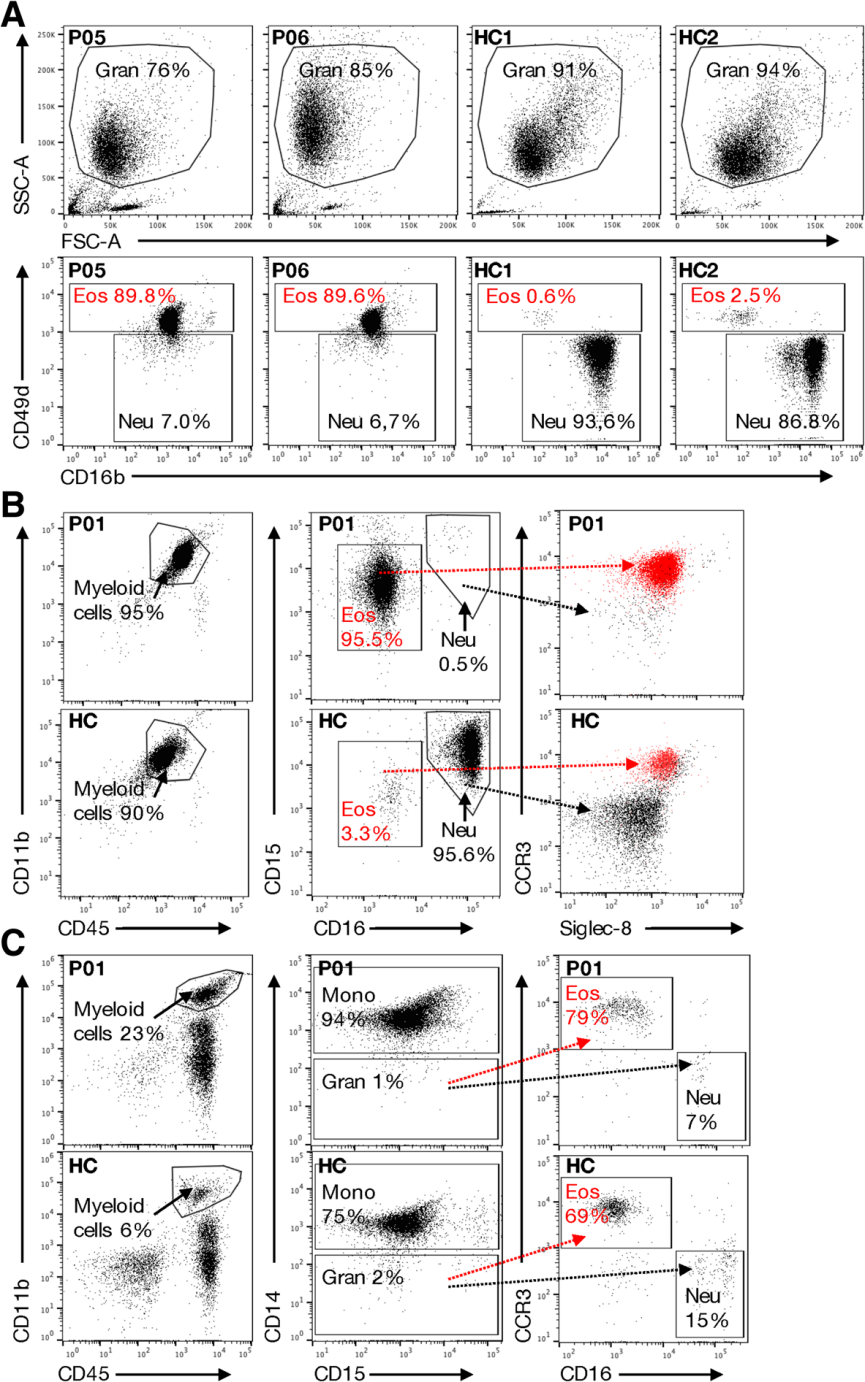
Eosinophils dominate Ficoll-Paque enriched SCN1 granulocyte fractions. Flow cytometry analysis was performed on (**a-b**) Ficoll-Paque enriched granulocytes (Gran) and (**c**) PBMCs to examine the number of neutrophils (Neu) and eosinophils (Eos). **a** Upper panel; Granulocytes from SCN1 patients (P05 and P06) and healthy controls (HC; *n* = 2) were gated based on side scatter (SSC) and forward scatter (FSC). Lower panel; The percentage of the different granulocytes (Eos; CD49d^+^CD16b^−^ and Neu; CD49d^−^CD16b^+^) are shown. **b** Granulocytes from one SCN1 patient (P01, upper panel) and one HC (lower panel) were gated for the percentage of myeloid cells (CD11b^+^CD45^+^), eosinophils (CD15^+^CD16^−^) and neutrophils (CD15^+^CD16^+^). The eosinophil population was confirmed by expression of CCR3 and Siglec-8 (shown in red) as compared to the CCR3^−^Siglec-8^−^ neutrophil population (shown in black). **c** The PBMCs from the same SCN1 patient (P01, upper panel) and HC (lower panel) were evaluated for the content of neutrophils by examining the percentage of myeloid cells (CD11b^+^CD45^+^), granulocytes (CD14^−^CD15^+^), eosinophils (CCR3^+^CD16^−^; red) and neutrophils (CCR3^−^CD16^+^; black)

Hereafter, the granulocytes were stained with mAbs that specifically recognize neutrophils and eosinophils. We used CD16b (expressed on neutrophils and basophils but not eosinophils), and CD49d (expressed on eosinophils but not on mature neutrophils) to classify cells isolated from two SCN1 patients. In agreement with the above results, a large proportion (~ 90%) of the SCN1 granulocytes were eosinophils (CD16b^−^CD49d^+^; Fig. 2a). In addition, isolated granulocytes from one SCN1 patients were also stained with a series of mAbs; CD45, CD11b, and CD15 that recognize both cell types, but also neutrophil-specific CD16 and eosinophil-specific Siglec-8 and CCR3. Our data show that the majority of CD45^+^CD11b^+^ granulocytes from SCN1 patients were CD15^+^CD16^−^. Further analysis of this population revealed high expression of CCR3 and Siglec-8, strongly signifying an enrichment of eosinophils with almost no neutrophils in granulocyte samples from the SCN1 patients (Fig. 2b). We also collected PBMCs and noticed that only 1% of the cells in the PBMCs were granulocytes (CD45^+^CD11b^+^CD14^−^), and that the majority of these (79%) were eosinophils (CD45^+^CD11b^+^; CD14^−^CD15^+^; CCR3^+^CD16^−^) and not neutrophils (Fig. 2c). Meanwhile, the percentage of the monocytes (CD45^+^CD11b^+^CD14^+^) were higher in the PBMCs of patients compared with those from controls (Fig. 2c), corroborating the data in Table 2. Taken together, these data support the blood smear-based cell counting (Fig. 1c) showing that eosinophils are the dominating granulocytes in the blood of SCN1 patients.

### Analysis of NADPH-oxidase activity in Ficoll-Paque enriched SCN1 granulocytes

**Fig. 3:**
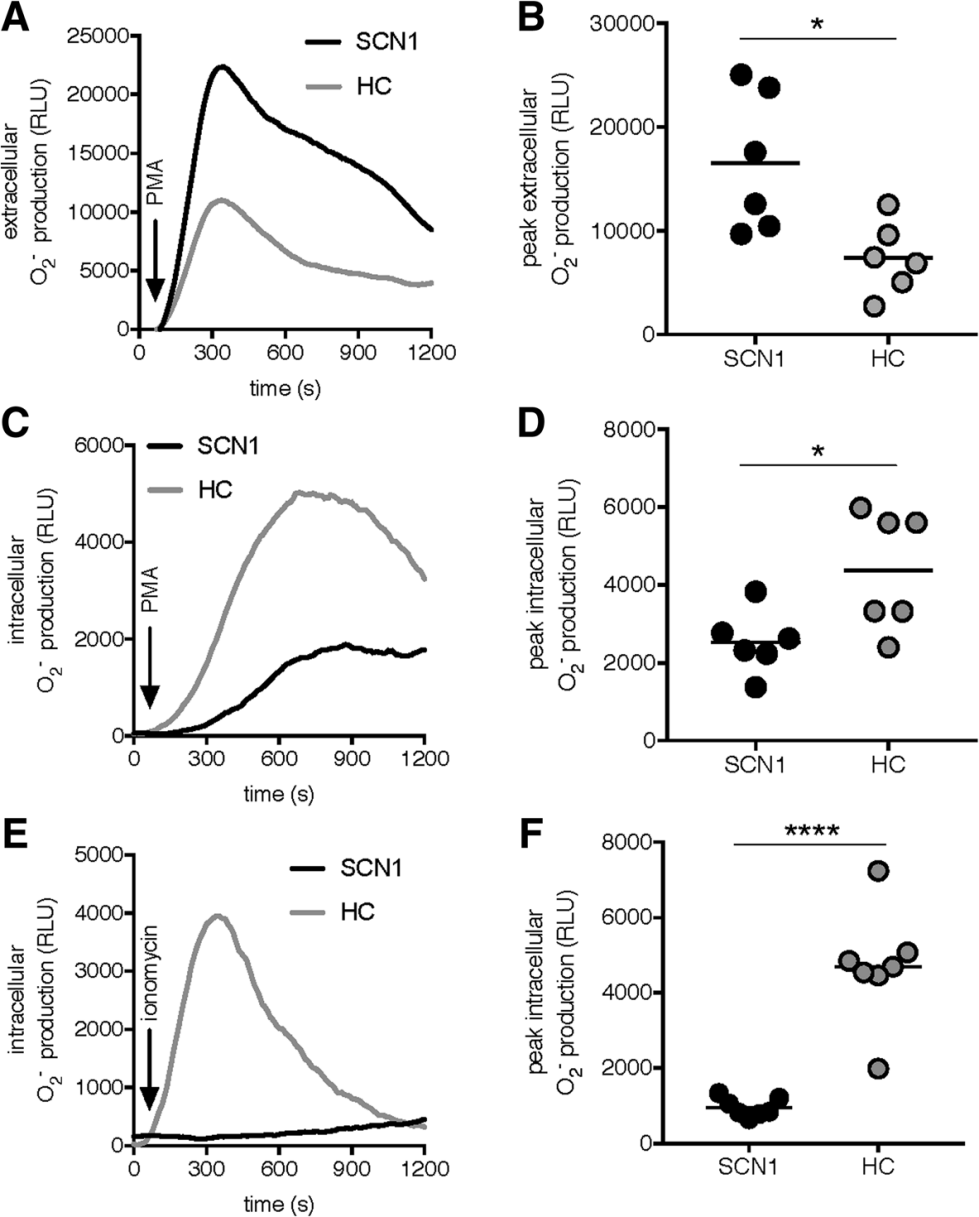
Diminished intracellular NADPH-oxidase derived ROS production in Ficoll-Paque enriched SCN1 granulocytes. ROS-production by granulocytes was measured in a plate reader using (**a-b**) isoluminol-amplified CL to measure extracellular ROS, and (**c-f**) luminol-amplified CL to measure intracellular ROS. The left lane shows one representative trace of ROS production (RLU) over time (seconds; s), and the right lane shows the peak ROS-production by SCN1 granulocytes (black line; black dots) and healthy control granulocytes (HC; grey lines; grey dots). Samples were stimulated with (**a-b**) PMA (50 nM, *n* = 6; P01, P03-P05, P07 and P09), (**c-d**) PMA (50 nM, *n* = 6; P01-P05 and P07), or (**e-f**) ionomycin (0.5 μM, *n* = 7; P01-P07). Statistical analysis in **b**, **d**, and **f** was performed by an unpaired Student’s *t*-test

**Fig. 4:**
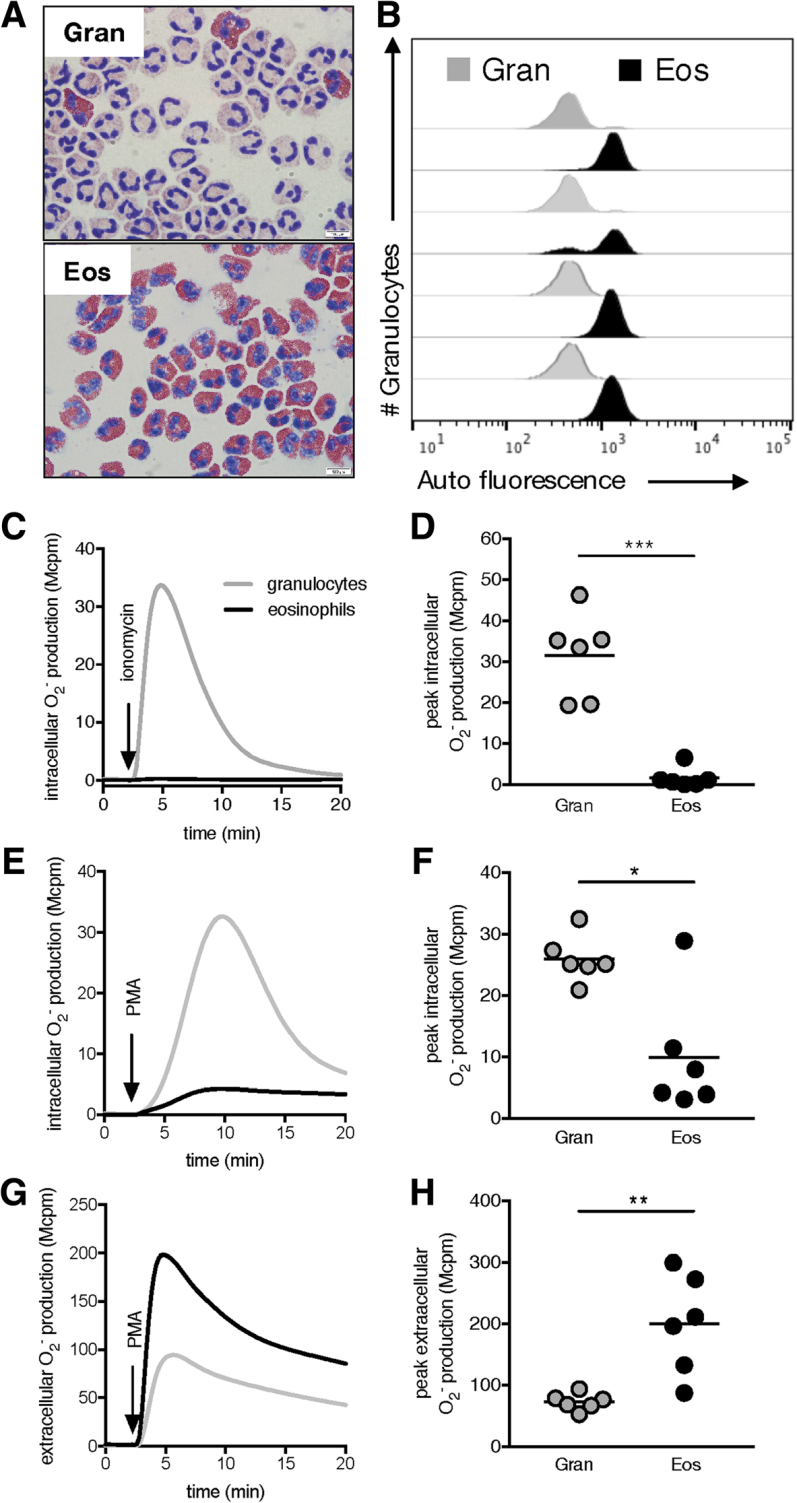
Diminished intracellular NADPH-oxidase derived ROS production by isolated eosinophils. Blood granulocytes from six healthy donors were isolated by Ficoll-Paque. Some granulocytes (Gran) were kept on ice and the rest was subjected to magnetic beads in order to isolate eosinophils (Eos) using magnetic beads. **a** Representative microscopic cytospin images, from one donor out of three, of granulocytes and purified eosinophils stained with May-Grünwald/Giemsa. **b** The histograms show the auto fluorescence of unstained eosinophils (black) as compared to unstained granulocytes (grey) in the FITC-channel as analysed by flow cytometry (*n* = 4). **c-h** ROS-production by granulocytes and isolated eosinophils from the same donor was measured on a Bioluminat LB9505 using (**c-f**) luminol-amplified CL to measure intracellular ROS, and (**g-h**) isoluminol-amplified CL to measure extracellular ROS. The left lane shows one representative trace of ROS production (Mcmp) over time (minutes; min), and the right lane shows the peak ROS-production (Mcpm) by isolated eosinophils (black line; black dots) or granulocytes (grey lines; grey dots) stimulated with (**c-d**) ionomycin (0.5 μM) and (**e-h**) PMA (50 nM). Statistical analysis in **d**, **f** and **h** was performed using a paired Student’s *t-*test after subtracting the stimulus induced peak-value to the value at time 0 min

**Fig. 5:**
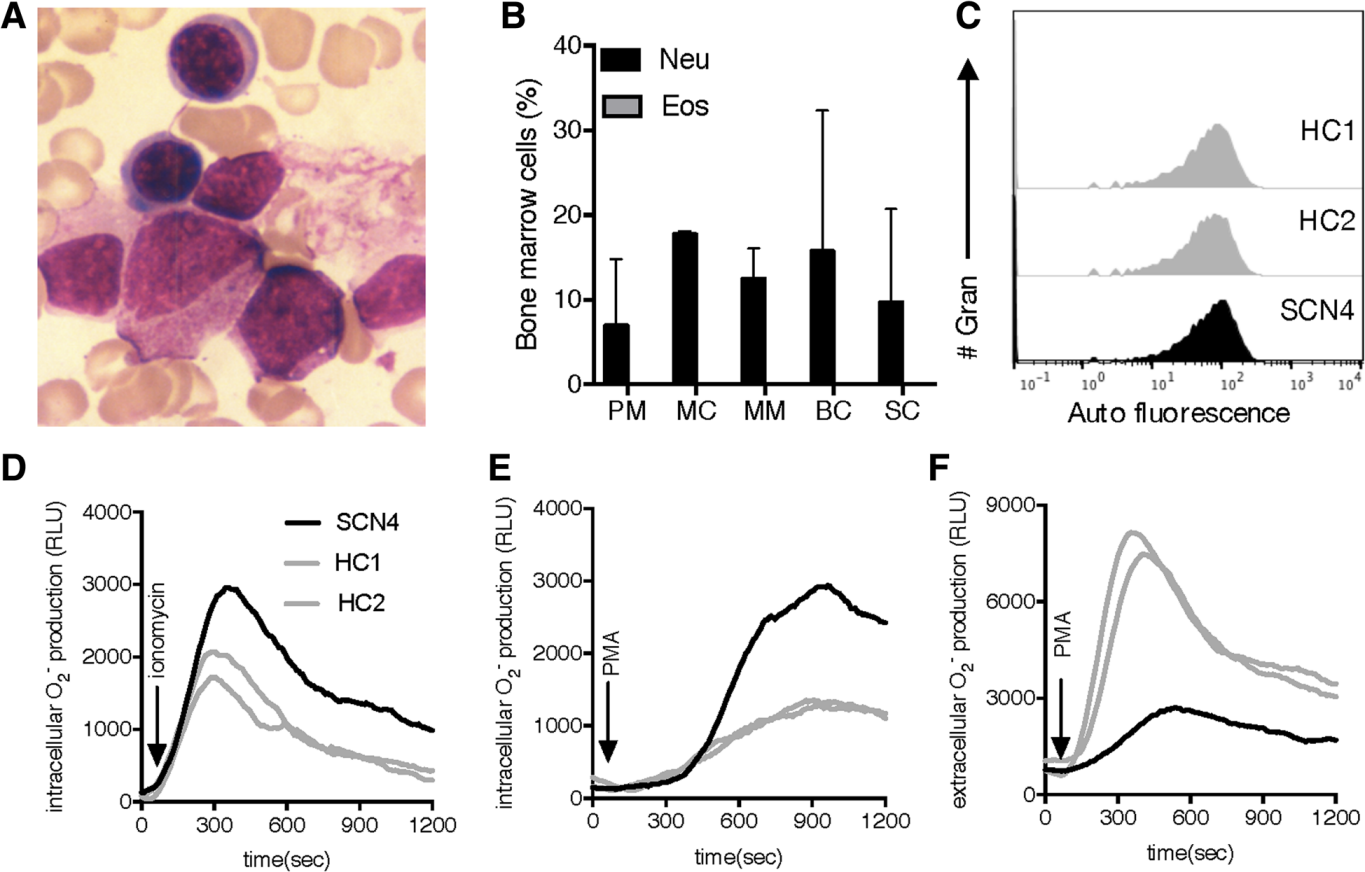
Characterization of bone marrow and blood cells from one SCN4 patient with heterozygous mutations in the *G6PC3* gene. **a** One representative image (out of 6) of bone marrow fluid stained with Wright’s stain from the SCN4 patient. **b** The bar graph shows the percentage (mean + SD; analysed at two separate occasions) of neutrophils (black bars) and eosinophils (grey bars) in different development stages in bone marrow samples; promyelocytes (PM), myelocytes (MC), metamyelocytes (MM), band cells (BC), and segmented cells (SC), from the SCN4 patient. **c** The histograms show the auto fluorescence of unstained Ficoll-Paque enriched granulocytes from two healthy controls (HC, grey), and the SCN4 patient (black). **d-f** NADPH oxidase derived ROS production by Ficoll-Paque enriched granulocytes from the SCN4 patient (black line) and two HC (grey lines) was measured in a plate reader using (**d-e**) luminol-amplified CL, and (**f**) isoluminol-amplified CL. The traces show (**d-e**) intracellular ROS production induced by (**d**) ionomycin (0.5 μM), (**e**) PMA (50 nM), and (**f**) extracellular ROS production induced by PMA (50 nM)

We next examined the function of the Ficoll-Paque enriched SCN1 granulocytes by measuring their capacity to generate the NADPH-oxidase derived ROS [19, 24]. Activation of granulocytes is associated with an increase in cellular consumption of molecular oxygen to generate highly reactive ROS through the NADPH-oxidase. ROS are not only strong bactericidal substances, but may also cause tissue damage and act as signaling molecules [19]. The Ficoll-Paque enriched SCN1 granulocytes released significantly higher amounts of extracellular ROS upon PMA stimulation (Fig. 3a-b), whereas the levels of PMA-induced intracellular ROS were significantly lower in SCN1 granulocytes as compared to that of the control granulocytes (Fig. 3c-d). Intracellular ROS production was further examined using ionomycin, a calcium ionophore that predominantly induces a translocation of cytosolic components of the NADPH-oxidase to the neutrophil granule membrane resulting in intracellular ROS production [25]. Our data clearly show that SCN1 granulocytes did not respond to ionomycin, unlike the control granulocytes (Fig. 3e-f). To examine if this was due to the fact that control granulocytes are dominated by neutrophils, and SCN1 granulocytes by eosinophils, we tested purified eosinophils obtained from healthy blood donors. The purified eosinophils, characterized by May-Grünwald/Giemsa staining (Fig. 4a), and auto fluorescence (Fig. 4b), also showed increased PMA-induced extracellular ROS production (Figs. 4c-d). In addition, purified control eosinophils displayed decreased PMA-induced intracellular ROS (Fig. 4e-f) and diminished ionomycin-induced intracellular ROS-production (Fig. 4g-h). That is, the ROS profile from purified control eosinophils was strikingly similar to the SCN1 granulocytes. In addition, the fact that ionomycin provoked normal intracellular ROS production in granulocytes isolated from the SCN4 patient (Fig. 5d) that primarily contained neutrophils (Fig. 5c), further supports the suggested difference regarding NADPH-oxidase activity between neutrophils and eosinophils. Also, when stimulating the SCN4 patients’ granulocytes with PMA the intracellular ROS was somewhat higher (Fig. 5e) and extracellular ROS somewhat lower (Fig. 5f) as compared to controls, i.e., in opposite to both SCN1 granulocytes (Fig. 3a-d) and purified eosinophils (Fig. 4e-h).

Taken together, these data not only confirm a dominance of eosinophils instead of neutrophils in the granulocyte population of SCN1 patients, but also show a normal NADPH-oxidase activity of SCN1 eosinophils.

## Discussion

In this study, we examined the composition and functionality of granulocytes obtained from nine SCN1 patients representing eight different *ELANE* gene mutations. Our data clearly demonstrate that the granulocyte population of SCN1 patients is dominated with functionally normal eosinophils, as revealed by several methods including blood smear examination, surface marker analysis, and functional studies. The clinical phenotype of the SCN1 patients were in accordance with previous reports including recurrent bacterial infections and a maturation arrest at the promyelocyte stage in the bone marrow [5, 6, 14]. Three of the patients suffered from *Mycobacterium tuberculosis* (TB) infection which may be explained by an increased prevalence of TB in China [26] and that neutrophil elastase is important for immunity against TB [27].

All our data point out that SCN1 granulocytes are dominated with eosinophils except the data obtained from automatic blood analysers showing much higher counts of neutrophils than eosinophils. Our data are in line with previous reports showing maturation arrest at the promyelocyte stage in association with hypereosinophilia in SCN1 [5, 6, 10, 14, 15]. However, our data obtained from one SCN4 patient indicate that increased eosinophils is not a general phenomenon for all types of SCN. For the SCN4 patient, as well as other non-neutropenia patients treated at the Children’s hospital, Chongqing Medical University in China, the data from automated blood analysers showed similar results as blood smear examinations. This is the first study showing a discrepancy regarding granulocyte composition between automated blood analysers and blood smears in SCN1 patients. The discrepancy may be explained by that SCN1 eosinophils also display minor maturation defects and by that the automated blood analysers mistakes these eosinophils for neutrophils. Thus, further characterization of the SCN1 eosinophil linage in the bone marrow is needed. Complete blood counts and white blood cell subset have traditionally been examined microscopically with blood smears. Yet, as automated blood analysers provide much faster results they are today the standard way of analysing complete blood counts for a variety of patients, including SCN1. For SCN1 patients, blood counts are critical for the judgment of disease severity and adjustment of the dosage for treatment with G-CSF. The results obtained in this study strongly recommend that blood smears should be performed in parallel to automated blood analysers when determining the ANC for SCN1 patients.

By using the Ficoll-Paque separation method, we recovered granulocytes from the peripheral blood of SCN1 patients. This separation method is very commonly used to enrich neutrophils from healthy blood donors as neutrophils are the dominating granulocytes in healthy control blood. However, as the granulocyte composition in SCN1 patients is skewed to eosinophils, their Ficoll-Paque enriched granulocyte population mainly contain eosinophils. We confirmed this by the feature of eosinophils being auto fluorescent in the FITC channel and by cell surface specific mAbs against neutrophils and eosinophils (Figs. 1 and 2). Further, functional analysis of the SCN1 granulocytes NADPH-oxidase activity supports the notion that they are eosinophils and not neutrophils. Thus, the classical Ficoll-Paque separation method should be avoided when investigating neutrophils from SCN1 patients as the neutropenia and hypereosinophilia in these patients skew the granulocyte composition to be dominated by eosinophils.

Our functional characterization of the SCN1 granulocytes, measured as NADPH-oxidase activity, demonstrate that these cells display a ROS production profile almost identical to that of purified control eosinophils. Apart from corroborating that the SCN1 granulocyte population is dominated by eosinophils, these data contribute with the novel fact that SCN1 eosinophils have a normal functional NADPH-oxidase which has not been reported before. Moreover, the NADPH-oxidase activation profile in the SCN4 granulocytes (dominated with neutrophils) was very similar to that of healthy control granulocytes. This further supports the notion that increased eosinophils is not a general feature for all types of SCN. NADPH-oxidase derived ROS are essential not only for bacterial killing, but also play a critical role in cell signaling and can, if not properly controlled, cause tissue damage. The precise mechanism of NADPH-oxidase assembly and activation in neutrophils and eosinophils is poorly understood, but our data showing a difference in ROS production between these two types of granulocytes support the fact that eosinophils and neutrophils have different roles in host defence. This implies that the increased eosinophils in SCN1 patients cannot fully compensate for the loss of neutrophils regarding the oxygen-dependent killing machinery.

It remains to be elucidated why SCN1 patients have increased eosinophils. Neutrophils and eosinophils are derived from a common myelocytic-committed progenitor, the myeloblast, but driven to maturation by different combinations of transcription factors, cytokines, and localized niches in the bone marrow [28, 29]. Structural perturbations of elastase protein with aberrant intracellular processing and trafficking are proposed to be the main cause behind the blockade of neutrophil maturation and induction of cell death in the bone marrow of SCN1 patients [30, 31]. However, how this could promote eosinophil progenitor differentiation is difficult to understand. Although we had access to only one SCN4 patient due to mutation of the *G6PC3* gene, our data derived from this patient clearly show that SCN4 has a different granulocyte composition from that of SCN1 patients. This thus suggests that increased eosinophil counts is not a general compensation mechanism for all types of SCN. This could depend on the underlying mechanism of the neutropenia, SCN4 is for example not always accompanied by a maturation arrest of neutrophil precursors [32]. It could also be due to a difference between neutrophil death in SCN1 and SCN4, as SCN4 has been associated with increased neutrophil apoptosis in addition to functional defects in macrophages [33, 34].

## Conclusions

Our in-depth characterization of SCN1 blood granulocytes clearly demonstrates a skewed granulocyte composition with increased eosinophils in circulation, which is evident by blood smear analyses but not by automated blood analysers. Accordingly, SCN1 granulocytes enriched by Ficoll-Paque isolation are dominated by eosinophils. The exact mechanism underlying the eosinophilia in SCN1 remains to be investigated. However, the data presented in this study shows functionally normal NADPH-oxidase activity by SCN1 eosinophils, that differs considerably from that of healthy control neutrophils. This indicates that the increased eosinophils in SCN1 patients cannot compensate for the loss of neutrophils regarding the NADPH-oxidase dependent microbial killing machinery.

## Data Availability

The data utilised in this article are available from the first authors (liuqiao_019@163.com and martina.sundqvist@rheuma.gu.se) upon reasonable request.

## Abbreviations

ANC: Absolute neutrophil count
BC: Band cells
CRP: C-reactive protein
Eos: Eosinophils
FITC: Fluorescein
G-CSF: Granulocyte colony stimulating factor
Gran: Granulocytes
HC: Healthy control
KRG: Krebs-Ringer phosphate buffer
mAbs: monoclonal antibodies
MC: Myelocytes
MM: Metamyelocytes
MPO: Myeloperoxidase
Neu: Neutrophils
PBMCs: Peripheral blood mononuclear cells
PM: Promyelocytes
PMA: Phorbol 12-myristate 13-acetate
ROS: Reactive oxygen species
SC: Segmented cells
SCN: Severe congenital neutropenia
SCN1: SCN due to *ELANE* gene mutations
SCN4: SCN due to *G6PC3* gene mutations
